# The impact of Parkinson’s Disease on interlimb coordination: a scoping review of gait adaptability

**DOI:** 10.3389/fpsyg.2025.1621770

**Published:** 2025-08-14

**Authors:** Morteza Farivar, Boglarka S. Huddleston, Adam C. King

**Affiliations:** ^1^Department of Kinesiology, Texas Christian University, Fort Worth, TX, United States; ^2^School of Medicine, Stanford University, Palo Alto, CA, United States; ^3^School of Medicine, Department of Medical Education, Texas Christian University, Fort Worth, TX, United States

**Keywords:** gait adaptability, Parkinson’s Disease, gait analysis, motor dysfunction, interlimb coordination, phase coordination index

## Abstract

**Introduction:**

Interlimb coordination, the synchronization of movements between limbs, is essential for efficient and stable human movement. Disruptions in coordination contribute to gait dysfunction, a common challenge for individuals with Parkinson’s disease (PD). This scoping review investigates how PD impairs interlimb coordination and influences gait adaptability, emphasizing the complexity of motor control challenges.

**Methods:**

This review assessed individuals with PD, focusing on spatiotemporal parameters and interlimb coordination within the Population, Concept, and Context (PCC) framework. A literature search was conducted across PubMed, Web of Science™, Scopus, SPORTDiscus, and Google Scholar™ in June 2023, following PRISMA-ScR guidelines. Risk of bias was assessed using an instrument proposed by Downs and Black (1998). Out of 710 studies, 14 met the inclusion criteria. Coordination was evaluated during treadmill or overground walking.

**Results:**

The review identified significant gait impairments in individuals with PD, including reduced walking velocity, step length, and range of motion. Coordination deficits were reflected in increased synchronization delays, phase shifts, and higher Phase Coordination Index values, particularly among those with freezing of gait. The findings emphasize the variability in PD’s motor effects and highlight the need for individualized assessments and targeted strategies to address gait dysfunction and coordination impairments.

**Discussion:**

This review highlights the critical impact of PD on gait dynamics and interlimb coordination, reinforcing the need for personalized interventions aimed at improving coordination, enhancing mobility, reducing fall risk, and improving quality of life.

## 1 Introduction

The successful navigation of daily life necessitates the constant adaptation of human movement to changing environments. Gait adaptability, the capacity to modify walking patterns in response to environmental demands or task constraints, is essential for safe and efficient locomotion (Hak et al., 2013; Weerdesteyn et al., 2018) and encompasses both reactive adjustments, such as recovering from a trip (Weerdesteyn et al., 2018), and proactive modifications like adapting to uneven terrain (Dixon et al., 2018; Ippersiel et al., 2021). Understanding the adaptive aspects of successful gait is crucial as it allows for examination of continuous, real-time adjustments in foot placement, step length, and interlimb coordination, which are essential for safely navigating complex environments and efficiently maintaining balance and forward progression (Caballero et al., 2019; Hafer and Boyer, 2018). Impaired adaptability observed in older adults and individuals with Parkinson’s Disease (PD) can lead to difficulties in avoiding obstacles, navigating uneven terrain, and responding to unexpected perturbations, ultimately increasing the risk of falls (Alcock et al., 2018; Blumen et al., 2020; Hak et al., 2013). For PD individuals, these challenges are particularly pronounced due to the disease’s broad impact on motor control properties that further compromises the ability to effectively adapt gait patterns (Cole et al., 2017; Huang et al., 2012; Lin and Wagenaar, 2018). Affecting 30 to 60% of individuals with PD annually, falls can result in traumatic brain injuries or hip fractures and have been associated with interlimb coordination impairments that compromise balance, recovery from perturbations, and gait adaptability (Ippersiel et al., 2021; Mainka et al., 2023; Rubenstein, 2006). Understanding how PD disrupts interlimb coordination is therefore essential for identifying the underlying mechanisms associated with fall risk and developing interventions to reduce fall risks and enhance gait adaptability.

Interlimb coordination – synchronized movement of two body segments – plays a pivotal role in enabling dynamic adjustments that ensure safe locomotion (Agurto et al., 2021; Peterson et al., 2012; Roemmich et al., 2013; Weersink et al., 2022). However, PD disrupts interlimb coordination, leading to impairments in the timing, rhythmicity, and synchronization of limb movements (Agurto et al., 2021; Arippa et al., 2022; Mainka et al., 2023; Weersink et al., 2022). These impairments are further exacerbated by the natural aging process, which also affects lower limb coordination and variability (Ippersiel et al., 2021; Mainka et al., 2023; Rodriguez et al., 2013). Thus, coordination analyses provide insight into how the nervous system regulates movement by quantifying the precision and stability of interlimb synchronization (Dietz, 2011; Hausdorff, 2007) while also helping to identify disruptions in neural control strategies associated with PD and aging (Israeli-Korn et al., 2019; Plotnik et al., 2008; Yogev et al., 2007).

Quantifying interlimb coordination in PD remains a challenge as different methodologies have been employed, leading to varying theoretical and practical insights. Previous evidence has focused primarily on spatiotemporal parameters like stride length and cadence (Agurto et al., 2021; Arippa et al., 2022; Filippin et al., 2020), while others utilize gait kinematics such as joint angles and range of motion (Agurto et al., 2021; Arippa et al., 2022; Filippin et al., 2020). Understanding the strengths and limitations of these diverse methodologies is crucial for selecting appropriate assessment tools and developing targeted interventions.

This scoping review provides a comprehensive inspection of the current evidence around how the coordination patterns of walking are impacted by PD. By synthesizing the current evidence, this review aims to: (1) examine how PD influences interlimb coordination; (2) identify the key methods used to assess interlimb coordination in individuals with PD; and (3) highlight the strengths and limitations of current assessment techniques to guide future research and clinical interventions. Ultimately, this review seeks to inform our current understanding of interlimb coordination in PD and others the potential to be incorporated into the development of effective interventions to improve gait adaptability, reduce fall risk, and enhance quality of life for individuals with this condition.

## 2 Methods

This scoping review was conducted in accordance with the Preferred Reporting Items for Systematic Reviewers and Meta-Analysis extension for Scoping Reviews (PRISMA-ScR) (Peters et al., 2020). Studies that focused on the impact of PD on interlimb coordination during gait were collected.

### 2.1 Search strategy

Electronic database searches were conducted that included Web of Science™, Scopus, PubMed, SPORTDiscus. Google Scholar served as a supplementary search platform due to its less formal application in identifying articles not retrievable through the primary four databases utilized. A comprehensive search strategy was implemented across all chosen databases using keywords and controlled vocabulary where available. The search string incorporated critical contextual terms for “Parkinson’s Disease,” “interlimb,” “coordination,” “gait,” and “gait variability” to facilitate the retrieval of relevant articles. The search was conducted in June 2023 and was limited to articles written in English but did not include limitation on publication date.

### 2.2 Inclusive and exclusion criteria

The eligibility criteria were predetermined and structured around the PCC (Population, Concept, Context) framework (Taylor et al., 2023). The Population of interest was individuals diagnosed with PD who were aged 55 years or older, reflecting the typical age of onset and progression of motor decline associated with the disease. The Concept under investigation was the assessment of interlimb coordination during continuous, straight-line walking, which has been identified as a critical aspect of motor control impacting individuals with PD and reflects the complexity of neurological dysfunction in gait dynamics. Lastly, the Context was defined as ‘open’, indicating a broad inclusion of settings where gait analysis might take place, encompassing both clinical and everyday environments. This approach ensured a comprehensive capture of relevant data and facilitated a nuanced understanding of interlimb coordination for individuals with neurological disorders like PD.

This review focused on observational studies, randomized controlled trials, and cross-sectional investigations that assessed gait characteristics in individuals with the neurological disorder of PD in comparison to older adults. Inclusion criteria for scoping review qualifications were studies reporting on: (1) assessments of straight-line overground walking or treadmill walking; (2) participants diagnosed with neurological disorders, specifically PD, in the “on” medication phase, irrespective of age, sex, and disease stage; (3) a comparison group of age- and sex-matched healthy individuals.

Studies were excluded that did not report on the required spatiotemporal gait variables or that lacked coordination metrics. Within the participant groups conditions such as essential tremor, postural deviations such as Pisa syndrome, individuals newly diagnosed with PD, and parkinsonism symptoms, were excluded as well as studies that report asymmetrical gait patterns or that were presenting duplicate data.

### 2.3 Source of evidence screening and selection

The selection process for identifying relevant evidence involved multiple stages. Two reviewers (M. F.; A. C. K.) conducted the selection of studies independently. Initially, titles and abstracts of studies identified via the search strategy were assessed against predefined eligibility criteria. In the second stage of screening, the selected studies underwent full-text evaluation by the same two independent reviewers to ensure adherence to the eligibility guidelines. Conflicts during the screening process were discussed and agreed mutually agreed upon after further discussion.

### 2.4 Data extraction

From the selected publications (*n* = 14), relevant information was extracted and categorized into data sets covering study characteristics, data collection and processing approaches, and coordination findings. Study characteristics included data related to author/year, study design, participant’s characteristics, walking conditions, while information pertaining to instruments, data processing and analysis, main spatial–temporal gait outcomes were included in the second batch of extracted data. The findings around the assessment and results of interlimb coordination were group together for table presentation. The same two reviewers who independently selected the studies undertook data extraction.

### 2.5 Analysis and presentation of results

The results of the screening and selection process are presented in table formation with descriptive summaries provided related to the study’s aim and questions.

The primary outcome measures were determined *a priori* and related to the spatiotemporal and coordination metrics of gait. Detailed measurements (i.e., means and standard deviations) of gait parameters such as spatiotemporal characteristics, walking distance, stride length, cadence, step width, phases of double and single support, swing time, and range of motion (ROM) for the hip, knee, and ankle joints during the gait cycle. The analysis of coordination offers an understanding of interlimb dynamics during gait and can be assessed using different metrics. A few of the common approaches to evaluating interlimb coordination involves examining phase shifts, synchronization delays, and phase coordination index (PCI), which collectively provide insights into the spatial–temporal organization of limb motion. For instance, Carpinella et al. (2007) and Crenna et al. (2008) discussed improvements in interlimb coordination through reduced phase shifts, indicating enhanced synchronization between limbs (Carpinella et al., 2007; Crenna et al., 2008). Similarly, Peterson et al. (2012) utilize PCI to assess the degree of bilateral coordination, revealing significant differences across PD subgroups and a correlation between freezing severity and coordination impairment (Peterson et al., 2012). Additionally, it is important to consider the plane of analysis with some studies examining multiple planes (Crenna et al., 2008; Lin and Wagenaar, 2018); however, sagittal plane analysis has been the predominantly focus when investigation gait (Carpinella et al., 2007; Crenna et al., 2008).

### 2.6 Risk of bias assessment

The methodological quality of the included studies was assessed using the Downs and Black checklist, a 27-item tool designed to evaluate both randomized and non-randomized studies (Downs, 1998). Other researchers have utilized this checklist with appropriately tailored questions (Hu et al., 2021; Zanardi et al., 2021). Since this review focused solely on observational studies, the checklist was adapted by removing items 22–27, as they were not applicable to this study type. The final version retained questions 1–3, 5–7, 9–21 that focused on external validity, bias, confounding, and power. All studies were independently assessed by two reviewers with conflicts resolved through discussion.

## 3 Results

### 3.1 Study characteristics

**Figure 1:**
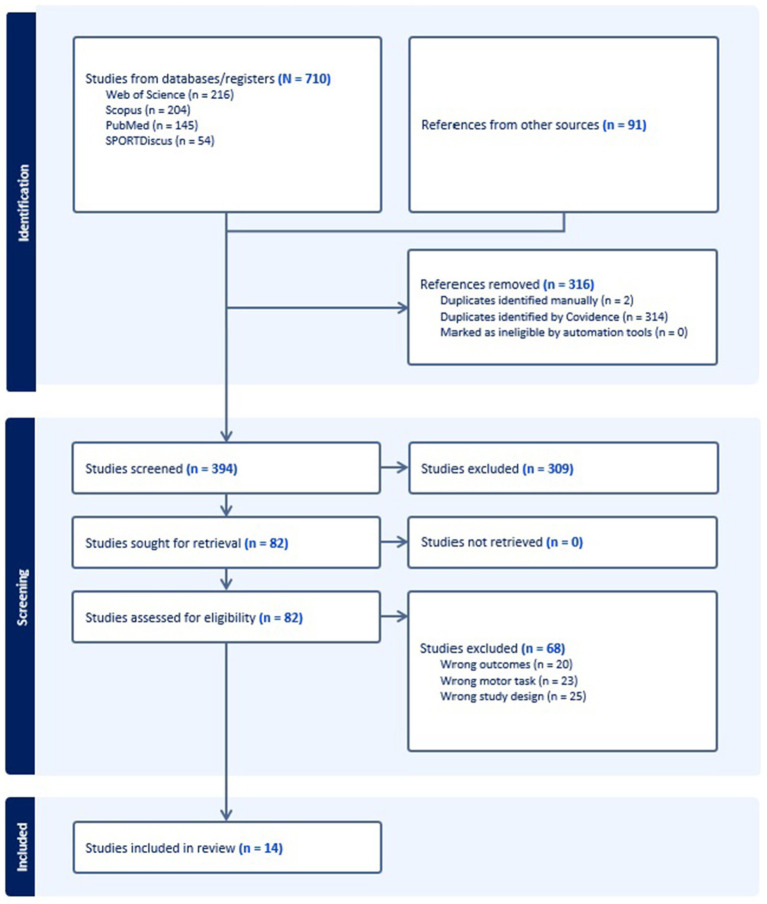
Flow diagram for the scoping review process adapted from the PRISMA statement.

The database search initially identified 710 studies. After removing 316 duplicate records, an additional 309 studies were excluded based on title and abstract screening. Full-text reviews were conducted for the remaining 82 studies, resulting in the exclusion of 68 studies due to (i) irrelevant outcome measures, (ii) inclusion of motor tasks other than straight-line gait, and (iii) study designs that did not align with the research objectives. Ultimately, 14 studies met the inclusion criteria. All included studies were cross-sectional, observational, and analytical in nature. The flow of articles through identification to final inclusion is represented in Figure 1.

### 3.2 Participant characteristics

Table 1 summarizes the participant characteristics across the selected studies. The sex distribution was approximately 40% female and 60% male, though three studies did not report this information. Participants’ ages ranged from the late 50s to late 70s, with an average age of 64.33 years in the PD group. Disease severity was assessed using the Hoehn & Yahr (H&Y) stage in eight studies (Agurto et al., 2021; Carpinella et al., 2007; Crenna et al., 2008; Filippin et al., 2020; Huang et al., 2012; Mainka et al., 2023; Nanhoe-Mahabier et al., 2011; Peterson et al., 2012) and the Unified Parkinson’s Disease Rating Scale (UPDRS) in nine studies (Agurto et al., 2021; Arippa et al., 2022; Huang et al., 2012; Mainka et al., 2023; Martínez et al., 2018; Nanhoe-Mahabier et al., 2011; Peterson et al., 2012; Roemmich et al., 2013; Tanahashi et al., 2013). While the H&Y stage provides a broad classification of disease progression based on motor impairment, the UPDRS offers a more detailed assessment of both motor and non-motor symptoms (Goetz et al., 2008).

**Table 1:** Descriptive participant’s characteristics and walking conditions descriptions.

Authors, year	Participant’s characteristics			Walking conditions
Carpinella et al. (2007)	PD group *N* = 10 (5F, 5 M) Age: 60.2 ± 4.8 Disease duration (years): 8–26 H&Y: 2.5–4.5 Time post-surgery: 3–28 months		Control group *N* = 12 Age (years): 61.4 ± 5	Overground walking Self-selected speed (PD and Control) 10 m walkway (8 trials) Control-2: 25 trials walking at different speed between 0.5 and 1.4 m/s (25 trials)
Crenna et al. (2008)	PD group *N* = 10 (5F, 5 M) Age (years): 60.2 ± 4.8 Disease duration (years): 8–26 H&Y: 2.5–4.5 Time post-surgery (months): 3–28 LEDD (mg): 75–500 mg/day Dopamine agonists: 0.54–15 mg/day		Control group *N* = 10 (4F, 6 M) Age (years): 61.4 ± 5	Overground walking Preferred (eight trials), slow (eight trials) and fast (eight trials) speeds, in random order 10 m path
Nanhoe-Mahabier et al., 2011	PD (Freezers) group *N* = 12 (4F, 8 M) Age 60.5 ± 7.9 MMSE: 29.3 ± 1.0 FAB: 16.0 ± 2.0 Disease duration (years): 9.6 ± 3.6 H&Y: 2.4 ± 0.3 UPDRS (Part III): 35.4 ± 8.9 NFOG-Q score (max. 24): 11.6 ± 5.3	PD (non-freezers) *N* = 15 (5F, 10 M) Age = 60.2 ± 9.2 MMSE: 29.1 ± 1.2 FAB: 15.7 ± 2.1 Disease duration (years): 7.7 ± 4.5 H&Y: 2.1 ± 0.3 UPDRS (Part III): 30.6 ± 7.0	Control group *N* = 15 (6F, 9 M) Age = 57.9 ± 7.3 MMSE: 29.4 ± 0.6 FAB: 17.3 ± 1.0	Overground and treadmill walking Self-selected speed 8 m walkway One min of walking on treadmill
Huang et al. (2012)	PD group *N* = 8 (7F, 1 M) Age (years): 63.2 ± 8.4 UPDRS III motor score: 10.5 ± 4.5 H&Y: 1.3 ± 0.5 Disease duration (months): 15.5 ± 13.1 LED (mg): 262 ± 179 mg		Control group *N* = 8 (7F, 1 M) Age (years): 62.1 ± 7.3	Overground walking Self-selected speed 8 m walkway
Peterson et al. (2012)	PD + FOG group *N* = 12 Age (years): 72 ± 9 Disease duration (years): 8.0 ± 4.5 UPDRS-3: 45.5 ± 15.2 H&Y: 2.63 ± 0.8 FOG-Q total score: 12.6 ± 4.1	PD – FOG group *N* = 19 Age (years): 71 ± 9 Disease duration (years): 6.6 ± 5.1 UPDRS-3: 41.6 ± 6.4 H&Y: 2.37 ± 0.40 FOG-Q total score: 4.2 ± 3.9	Control group *N* = 10 Age (years): 69 ± 11	Overground walking Self-selected speed Forward walking, backward walking, turning to the left and right 10 m walkway (forward walking, backward walking)
Rodriguez et al. (2013)	PD group *N* = 15 Age (years): 66.6 ± 7.8 Height (cm): 172 ± 9.5 Weight (kg): 80.2 ± 13.6		Healthy group *N* = 14 Age (years): 66.2 ± 7.1 Height (cm): 166 ± 13.3 Weight (kg): 69.7 ± 17.8	Treadmill (split-belt) walking Self-selected speed 10 min
Roemmich et al. (2013)	PD group *N* = 18 Age (years): 63.5 ± 8.93 Weight (kg): 76.5 ± 13.7 Height (m): 1.69 ± 0.09 Overground gait speed: 1.14 ± 0.12 m/s UPDRS motor score: 22.7 ± 7.38		Healthy group *N* = 15 Age (years): 63.7 ± 8.29 Weight (kg): 74.1 ± 14.7 Height (m): 1.70 ± 0.11 Overground gait speed: 1.20 ± 0.11 m/s UPDRS motor score: 22.7 ± 7.38	Overground walking Self-selected speed 8 m walkway
Tanahashi et al. (2013)	PD – FOG *N* = 11 (7F, 4 M) Age (years): 69.26 ± 6.0 Disease duration (years): 6.16 ± 5.0 UPDRS III score Total: 31.16 ± 11.1 Axial: 5.06 ± 1.5 Upper limb movement: 9.46 ± 3.7 Lower limb movement: 2.46 ± 2.0 Rigidity: 7.56 ± 4.4 Tremor: 2.56 ± 1.8		FOG without PD *N* = 9 (7F, 2 M) Age (years): 72.96 ± 5.8 Disease duration (years): 2.76 ± 0.8 FOG onset (years): 1.06 ± 1.0 UPDRS III score Total: 18.66 ± 6.8 Axial: 5.76 ± 1.7 Upper limb movement: 5.46 ± 4.3 Lower limb movement: 2.86 ± 1.5 Rigidity: 1.16 ± 0.8 Tremor: 0.36 ± 0.7	Overground walking Self-selected speed 20 m walkway
Lin and Wagenaar (2018)	PD group *N* = 24 (4F, 20 M) Age (years): 62.2 ± 9.7 MMSE score: ≥24		Healthy group *N* = 26 (12F, 14 M) Age (years): 72.3 ± 5.6	Treadmill walking 0.22 m/s 1.30 m/s 0.22 m/s One min of walking on treadmill
Martínez et al. (2018)	PD group *N* = 11 (1 F, 10 M) Age (years): 57 ± 7.84 Weight (kg): 81 ± 12.92 Height (m): 1.75 ± 0.09 Disease duration (years): 4 ± 1.8 UPDRS III score: 15 ± 4.28 LEDD (mg): 400 ± 327 mg MMSE: 29 ± 1.63		Healthy group *N* = 4 (4F, 0 M) Age (years): 56.5 ± 12.4 Weight (kg): 70 ± 11.03 Height (m): 1.70 ± 0.07 Young group *N* = 16 (7F, 9 M) Age (years): 29.5 ± 3.63 Weight (kg): 68 ± 9.28 Height (m): 1.68 ± 0.08	Overground walking Self-selected speed 25 m walkway
Filippin et al. (2020)	PD group *N* = 10 (3F, 7 M) Age (years): 66.3 ± 9.37 Weight (kg): 68.65 ± 18.81 Height (m): 1.62 ± 0.09 MMSE: 27.1 ± 2.4 Disease duration (years): 6.5 ± 4.7 H&Y: 2.8 ± 0.35 Disease Rating Scale: 21.3 ± 4.19		Healthy group *N* = 10 (3F, 7 M) Age (years): 66.1 ± 9.21 years Weight (kg): 69.9 ± 9.94 Height (m): 1.65 ± 0.09 MMSE: 28.3 ± 1.8	Overground walking Self-selected speed 10 m walkway
Agurto et al. (2021)	PD group *N* = 33 (17F, 16 M) Age (years): 69 ± 8 Weight (kg): 84 ± 24 Height (cm): 171 ± 9 Dominant hand (%right): 90% Disease duration (years): 6 ± 4 LEDD (mg): 380 ± 304 mg UPDRS part III (ON/OFF): 40 ± 17/54 ± 16 UPDRS Gait (ON/OFF): 1.03 ± 0.95/1.45 ± 0.97 UPDRS Posture Stability (ON/OFF): 1.36 ± 1.11/1.76 + 0.90 H&Y: 2.15 ± 0.51 Clinical symmetry (Right/Left/None): 13/18/3 Asymmetry index: |L-R|/(L + R): 0.19 ± 0.17		Healthy group *N* = 31 (10F, 21 M) Age(years): 49 ± 9 Weight (kg): 79 ± 18 Height (cm): 175 ± 10 Dominant hand (%right): 88%	Overground walking Self-selected speed 10 m walkway
Arippa et al. (2022)	PD group *N* = 61 (24F, 37 M) Age (years): 68.9 ± 9.3 Weight (kg): 67.1 ± 10.9 Height (cm): 164.5 ± 7.8 Disease duration (years): 7.7 ± 5.6 UPDRS III score: 19.9 ± 9.3		Control group *N* = 47 (19F, 28 M) Age (years): 66.0 ± 8.3 Weight (kg): 66.9 ± 11.1 Height (cm): 164.7 ± 6.9	Overground walking Self-selected speed 10 m walkway
Mainka et al. (2023)	PD group *N* = 36 (19F, 17 M) Age (years): 61.7 ± 7.3 Weight (kg): 76.6 ± 16.2 Height (cm): 171.8 ± 10.8 Disease duration: 4.8 ± 3.4 years H&Y: 2.0 ± 0.6 UPDRS III motor score: 17.6 ± 7.7 LEDD (mg): 715.5 ± 265.6		Healthy group *N* = 36 (17F, 15 M) Age (years): 64.5 ± 9.0 Weight (kg): 78.8 ± 13.9 Height (cm): 172.1 ± 10.3:	Overground walking Very slow, slow, preferred, fast, and very fast 40 m walkway in gait speed

MMSE, Mini Mental State Exam; UPDRS, Unified Parkinson’s Disease Rating Scale; H&Y, Hoehn & Yahr; FAB, Frontal Assessment Battery; NFOG-Q, New Freezing of Gait Questionnaire; LED, Levodopa Equivalent Dose; LEDD, Levodopa Equivalent Daily Dose.

Given the strong influence of dopaminergic therapy, it is also critical to consider the reporting of the medication state for PD individuals during testing when interpreting gait and coordination outcomes. Thirteen studies reported medication status, with the majority of assessments conducted in the “ON” medication state, typically between 60 min to 12 h post-medication. Only one study (Tanahashi et al., 2013) conducted assessments in the “OFF” state, allowing for a reasonably consistent interpretation of coordination metrics across medication contexts. Cognitive function was assessed using the Mini-Mental State Examination (MMSE) in three studies (Filippin et al., 2020; Martínez et al., 2018; Nanhoe-Mahabier et al., 2011) with scores ranging from 27.1 to 29.4, with an average score of 28.5, indicating relatively preserved cognitive function among participants.

### 3.3 Walking conditions

The walking conditions analyzed in this scoping review were selected to examine gait and mobility challenges in individuals with PD. Eleven studies assessed interlimb coordination during overground walking at self-selected gait speeds (Agurto et al., 2021; Arippa et al., 2022; Carpinella et al., 2007; Crenna et al., 2008; Filippin et al., 2020; Huang et al., 2012; Mainka et al., 2023; Martínez et al., 2018; Peterson et al., 2012; Roemmich et al., 2013; Tanahashi et al., 2013), while two studies focused on treadmill walking (Lin and Wagenaar, 2018; Rodriguez et al., 2013). One study incorporated both treadmill and overground walking, with participants walking at self-selected speeds in both conditions (Nanhoe-Mahabier et al., 2011). The walking distances for overground gait varied across studies, with most using a 10-meter walkway, some opting for an 8-meter walkway (Nanhoe-Mahabier et al., 2011), and others extending the distance beyond 20 meters (Mainka et al., 2023; Tanahashi et al., 2013).

### 3.4 Instruments

Table 2 presents the instruments used across studies to capture kinematic data used to examine interlimb coordination. Eight studies employed motion capture systems with a standard marker set, emphasizing kinematic analysis (Arippa et al., 2022; Carpinella et al., 2007; Crenna et al., 2008; Filippin et al., 2020; Lin and Wagenaar, 2018; Nanhoe-Mahabier et al., 2011; Rodriguez et al., 2013; Roemmich et al., 2013). In contrast, others introduced accelerometers and gyroscopes indicating a shift toward more portable and versatile measurement tools, which was complemented by the adaption of specialized like in-shoe pressure measurement system and digital cameras (Agurto et al., 2021; Huang et al., 2012; Mainka et al., 2023; Martínez et al., 2018; Tanahashi et al., 2013).

**Table 2:** Instruments, main outcomes and significant findings to assess interlimb coordination during gait.

Author, year	Instruments (Treadmill, motion capture, markers)	Data processing (filtering)	Data analysis	Main finding
Carpinella et al. (2007)	Kinematic recording: Motion Capture Marker set: 17	Sample Frequency: 50 Hz Filtering: low-pass filtered (cut-off frequency 3–7 Hz)	Spatiotemporal Gait speed ROM Upper arm angle Thigh angle	Gait Speed Significantly lower velocity in PD ROM Increase upper arm and thigh ROM in controls Significant reduction of upper arm and thigh ROM in PD Similar Upper Arm ROM Across Conditions (S+/M−, S−/M+)
Crenna et al. (2008)	3D kinematic gait analysis Marker set: 17	Sample Frequency: 50 Hz Filtering: low-pass filtered (cut-off frequency 3–7 Hz)	Spatiotemporal Gait speed ROM Absolute arm angle Absolute thigh angles Range of trunk torsion.	Walking Speed Significantly lower in PD patients compared to controls Arm and Thigh ROM: Significantly lower arm and thigh ROM in PD compared to controls Smaller ROM with increased walking speed in PD compared to controls.
Nanhoe-Mahabier et al. (2011)	Kinematic recording: Motion Capture (VICON) Marker set: NA Plug-in-Gait marker set	Sample frequency: NA Filtering: NA	Spatiotemporal Gait speed Spatial variability Temporal Variability Spatial Asymmetry Temporal Asymmetry	Gait velocity Larger preferred speed during overground walking compared to treadmill Higher gait speed in controls than freezers during treadmill and overground walking Spatial step regulation Larger step length in controls compared to freezers for both conditions Larger step length in non-freezers than freezers during treadmill walking Higher step variation in PDs than controls during treadmill walking Temporal step regulation Larger step time in freezers compared to controls during treadmill walking Larger step time asymmetry in non-freezers compared to controls during treadmill walking
Huang et al. (2012)	Kinematic recording: Accelerometer Sensor set: 2 for each arm	Sample frequency: 512 Hz Filtering: 50 Hz using a 3rd order Butterworth filter	Spatiotemporal Bilateral: Arm swing asymmetry Maximal cross-correlation Angular accelerations	Arm swing asymmetry in PD Significant differences in forearm accelerations Less symmetry and a more chaotic appearance Reduced arm swing amplitude Higher arm swing asymmetry and lower coordination Maximal cross-correlation MXC-ASA correlation varies, indicating inconsistent movement-asymmetry link in PD
Rodriguez et al. (2013)	Kinematic recording: Motion Capture Marker set: 35	Sample frequency: 120 Hz	Spatiotemporal Gait speed	Walking speed Lower in PD, but the difference was not statistically significant
Roemmich et al. (2013)	Kinematic recording: Motion capture Marker set: 35	Sample frequency: 120 Hz Filtering: NA	Spatiotemporal Walking speed ROM Hip joint Shoulder joint	Walking speed No significant difference between PD and controls ROM Significantly reduced in more affected hip in PD Not reduced in less affected hip or shoulders compared to controls
Tanahashi et al. (2013)	Kinematic recording: Accelerometers/Gyroscopes Sensor set: NA	Sampling Frequency: 100 Hz Filtering: NA	Spatiotemporal Stride time Step time Swing time Stance time	Gait parameters variability FOG–P exhibited less rigidity than PD–FOG. FOG–P showed more gait variability and hesitation; PD–FOG had more stable stride time and less deviation in step phase High stride time variability in FOG–P Gait Parameter Stability PD–FOG exhibited relatively stable step phase and stride time during straight walking, despite swing time asymmetry and deviation from 180° step phase
Lin and Wagenaar (2018)	Kinematic recording: 3D kinematic data with Optotrak 3020 System Marker set: 19	Sample Frequency: 100 Hz Filtering: NA	Spatiotemporal Leg Swing Angle Arm Swing Angle Pelvic Rotation	Kinematics Larger mean ӨLALL in older adults than younger Decreased in Group-Speed Interaction (ӨRALA Variability) with increasing speed; larger in PD than HC (except at slowest speed). Larger Gender Effect (ӨRLLL SD) in males than females. Increased Group-Speed Interaction (Mean ӨPT) with speed; higher in HC than PD at all speeds; group difference widens with speed Larger Age Effect (SD of ӨPT) in elderly than younger participants
Martínez et al. (2018)	Kinematic and Kinetic recording: F-scan in-shoe pressure measurement system Sensor set: NA	Sample Frequency (Kinetic): 100 Hz Filtering: NA	Spatiotemporal Stride time Stance time Swing time PST PSWT DST Stride-to-stance	Kinematics **S**tride Time similar across groups in both legs for all groups Similar DST average across groups; higher DST CV in PD than YC and AMC Increased Gait Asymmetry (GA) in PD and AMC compared to YC
Filippin et al. (2020)	Kinematic recording: digital cameras Markers set: 5	Sampling Frequency: 60 Hz Filtering: Fourth-order Butterworth filter with a cut-off frequency of 10 Hz	Spatiotemporal Stride length Speed Cadence Stride duration Stance phase duration Swing phase duration Stance Swing	Spatiotemporal differences Shorter stride length, slower speed, reduced cadence in PD Longer stride duration, stance, and swing phases in PD Similar stance and swing phase durations as percentage of gait cycle Joint angle variations Smaller ROM in Study group Ankle: Less plantar flexion at toe-off Knee: Smaller flexion during stance and swing; earlier flexion peak at initial contact Hip: Less flexion at start/end of gait cycle; smaller extension during pre-swing
Agurto et al. (2021)	Kinematic recording: Opal Version 1 wearable sensors (APDM Wearable Technologies) Sensor set: 6	Sampling Frequency: 128 Hz Filtering: 2nd order band-pass Butterworth filter with low cut-off frequency = 1 Hz and high cut-off frequency = 10 Hz	Spatiotemporal acceleration, angular velocity	PD severity estimation Acceleration signal correlated with UPDRS Velocity profile showed slightly better correlation Combined velocity and acceleration profiles increased correlation (right wrist/right-foot steps) Left–Right Body Movement Symmetry in PD In ON state: similar movement patterns in both feet In OFF state: distinct movement patterns, with greater differences in left foot Correlation with motor impairment Symmetry of steps and core (trunk and sternum) correlated with motor impairment scores Lumbar and trunk movements during left/right steps also significant for disease severity estimation Weaker correlation for arm swing symmetry
Arippa et al. (2022)	Kinematic recording: Motion Capture Marker set: 22	Sample Frequency: 120 Hz Filtering: NA	Spatiotemporal Speed Cadence (steps/min) Step Length Step Width Stance Phase Swing Phase Double Support Phase Dynamic ROM Hip, Knee, and Ankle ROM	Spatiotemporal parameters of gait: Reduced speed, step length, swing phase duration; increased double support phase duration compared to unaffected individuals Significantly higher SI Values in PD for double support and step length parameters Dynamic ROM Significantly reduced at hip and knee joints in PD compared to CG
Mainka et al. (2023)	Kinematic recording: APDM Mobility Lab (ML) system (version 2) Sensor set: 6	Sampling Frequency: 128 Hz Filtering: NA	Spatiotemporal: Cadence Gait velocity Stride Stride time variability Dynamic ROM Pelvis ROM Sternum ROM AS ROM AS peak angular velocity AS regularity	Spatiotemporal parameters of gait No significant differences in cadence, gait velocity, and stride between HS and PD across all five walking speeds Increased stride time variability in very slow condition in PD No statistical difference in cadence Arm swing and ROM A significant difference between both groups in AS and Higher asymmetry of AS in PD A lower mean ROM and peak angular velocity in normal, fast, and very fast walking

ROM, Range of Motion; S, Stimulation; M, Medication; FOG, Freezing of Gait; RALA, Right Arm and Left Arm; LALL, Left Arm and Left Leg; HC, Health Control; AS, Arm Swing; DST, Double Support Time; CV, Coefficient of Variation; AMC, Age-Matched Control; YC, Young Control; PST, Percentage of Stance Time; PSWT, Percentage of Swing Time; CG, Control Group; SI, Symmetry Index.

### 3.5 Spatiotemporal variables

A synthesis of findings illustrates key differences in gait dynamics between individuals with PD and healthy controls. Five investigations have reported that gait velocity was significantly lower in PD patients compared to controls, highlighting a fundamental compromise in mobility (Arippa et al., 2022; Carpinella et al., 2007; Crenna et al., 2008; Filippin et al., 2020; Nanhoe-Mahabier et al., 2011). In contrast, three studies observed non-significant differences, suggesting variability in the disease’s progression or the influence of compensatory mechanisms in some individuals (Mainka et al., 2023; Rodriguez et al., 2013; Roemmich et al., 2013).

Additionally, Nanhoe-Mahabier et al. (2011) noted a marked reduction in step length, particularly among freezers, affecting the safety of walking (Nanhoe-Mahabier et al., 2011). This finding was further supported with additional studies reporting shorter stride lengths in PD patients that could increase the risk of falls (Arippa et al., 2022; Filippin et al., 2020). Furthermore, the same studies indicated prolonged step times during treadmill walking, possibly, as an adaptive measure to maintain balance, yet cadence was notably reduced, signaling a general slowing of movement. Lastly, Martínez et al. (2018) and Arippa et al. (2022) found no significant differences in double support time between PD and control groups but observed a higher variability in PD patients, which might reflect a more unstable gait (Arippa et al., 2022; Martínez et al., 2018). These findings related to spatial temporal characteristics collectively underscore the impact of PD-related gait impairments and the importance of individualized assessment in clinical and therapeutic settings.

The information related to the range of motion (ROM) in patients with PD, including variations in upper arm and thigh ROM, the impact of walking speed, and asymmetry in joint mobility, is summarized in Table 2.

### 3.6 Interlimb coordination

Research on interlimb coordination in PD populations has examined synchronization delays between limb movements using various assessment methods. This information is detailed in Table 3. Nanhoe-Mahabier et al. (2011) and Huang et al. both observed larger synchronization delays between the more affected (MA) leg versus the MA arm and the less affected (LA) leg versus the LA arm in PD patients compared to controls during treadmill walking (Huang et al., 2012; Nanhoe-Mahabier et al., 2011).

**Table 3:** Summary of interlimb coordination findings categorized by joint segments, planes of movement, and corresponding coordination metrics.

Author, year	Plane of movement joint/segment	Main/significant findings to assess interlimb coordination
Carpinella et al. (2007)	Plane Sagittal Joint/Segment Upper body Lower body Coordination Phase shift	Interlimb coordination Significant higher phase shift between upper arm and thigh angles Reduction in Phase Shift Enhances Interlimb Coordination (S+/M-, S−/M+)
Crenna et al. (2008)	Plane Sagittal Horizontal Joint/Segment Upper body Lower body Coordination Phase shift	Interlimb coordination Significantly reduced the phase-shift between arm and ipsilateral leg motion in PD undergoing STN stimulation Significantly reduce the interlimb phase-shift in M + condition Significantly improved the interlimb phase-shift with S + M+
Nanhoe-Mahabier et al. (2011)	Plane Sagittal Joint/Segment Arm Heel Coordination Phase Coordination Index Ipsilateral synchronization Contralateral synchronization	Interlimb coordination Higher PCI in PD Ipsilateral synchronization Larger synchronization delay of MA leg vs. MA arm in non-freezers and freezers compared to controls during treadmill walking Larger synchronization delay of LA leg vs. LA arm in freezers and freezers compared to controls during treadmill walking, and tended to be larger in non-freezers compared to controls No differences between groups during overground walking, and no differences between freezers and non-freezers during overground walking Significant group condition interaction effect Contralateral synchronization Larger delay synchronization of MA leg vs. LA arm in freezers compared to controls (treadmill: *p* = 0.009; overground: *p* = 0.010) Larger in non-freezers compared to controls (treadmill: *p* = 0.017; overground: *p* = 0.019) Larger delay synchronization of LA leg vs. MA arm in freezers (*p* = 0.006) compared to controls during treadmill walking Larger in non-freezers compared to controls (*p* = 0.022) No differences between freezers and non-freezers
Huang et al. (2012)	Plane Sagittal Joint/Segment Forearms Coordination Instantaneous Relative Phase (IRP)	Interlimb coordination Highly significant differences between the IRP distributions Greater IRP variability among PD subjects
Peterson et al. (2012)	Plane Sagittal Joint/Segment Heel Coordination PCI	Interlimb coordination Smallest PCI values during forward walking Highest PCI values in PD + FOG subject Significantly higher PCI in PD + FOG than PD – FOG Significantly higher PCI in PD - FOG than controls
Roemmich et al. (2013)	Plane Sagittal Joint/Segment Shoulder Hip High/pelvis Upper arm/thorax Coordination CCC0 and CCC Max	Interlimb coordination Ipsilateral cross-correlation coefficients (CCC0 and CCC Max): Significantly reduced in PD (both less and more affected sides) compared to controls More pronounced reduction on the more affected side Contralateral CCC0 and CCC max: Significant reduction in less affected hip/more affected shoulder in PD No significant reduction in more affected hip/less affected shoulder compared to controls. Significant reduction in less affected hip/more affected shoulder in PD
Tanahashi et al. (2013)	Plane Sagittal Joint/Segment Heel Coordination: CV of step phase (QCV)	Interaction between group and walking condition: Significant for Δ*φ*_error, with larger Δφ_error during ‘Back’ in FOG–P No significant difference in PD–FOG between walking conditions Larger variability in stride time and step phase than PD–FOG, even if the step phase was closer to 180 than in PD–FOG. Large the deviation of step phase from 180 in FOG–P patients More forceful and noisier the phase correction in FOG–P than in PD–FOG
Lin and Wagenaar (2018)	Plane Sagittal Transverse Joint/Segment Shoulder Pelvic Coordination Relative phase	Interlimb coordination: Increased Variability in relative phase between left and right arm swing in PD individuals. Smaller Amplitude with arm and leg movements in PD. Less Variability in phase relation between thoracic and pelvic rotations in PD individuals.
Martínez et al. (2018)	Plane Sagittal Joint/Segment Heel Coordination PCI	Phase coordination index (PCI) Close PCI to ideal 180° in all groups; increased φ deviation in PD compared to young controls, not AMC Similar PCI in AMC and higher in PD compared to young controls; significant differences between PD and AMC
Filippin et al. (2020)	Plane Sagittal Joint/Segment Ankle Knee Hip Coordination Angle–angle plots: knee–ankle hip–ankle hip–knee The maximum cross-correlation coefficient Time lag between the joint pairs Bilateral evaluation	Intralimb coordination similarities: Angle–angle plots show similar angular displacements for knee–ankle, hip–ankle, and hip–knee across all gait events. Ankle plantarflexion affects knee–ankle and hip–ankle plots in the study group at gait cycle’s start/end. Relative joint movements (precedence/succession) similar across all gait phases. Cross-correlation and temporal coupling: Knee–ankle and hip–ankle segments inversely related (negative coefficients). Strong temporal coupling in control, moderate in study group. Hip–knee relationship shows strong coupling, movements in the same direction (positive coefficients). Interlimb coordination: No significant intergroup differences in phase values between right and left limbs (*p* = 0.36). Phase values near the ‘ideal’ 180°, indicating synchronized limb movements.
Arippa et al. (2022)	Plane Sagittal Joint/Segment the hip joints the knee joints the ankle joints Coordination Cyclogram area Angle-angle orientation	Point-by-point analysis of kinematic curves: significant differences between PD and CG at: the hip joints the knee joints the ankle joints Waveform-based symmetry indexes: Hip Joint Range Offset: Significantly larger in PD compared to unaffected individuals. Ankle Joint Symmetry Measures: Cyclogram orientation and Trend Symmetry significantly different in PD
Mainka et al. (2023)	Plane Sagittal Joint/Segment the fifth lumbar vertebrae sternum metatarsus wrist dorsally Coordination: AS coordination of arms	AS coordination in PWPD: Significant decreases in reciprocal timing during very slow and normal walking PD group’s coordination nears healthy levels in fast and very fast walking Improvement in AS coordination with increasing velocity, reaching values close to HS in fast and very fast walking

AS, Arm Swing; HS, Health Subjects; DST, Double Support Time; PCI, Phase Coordination Index; AMC, Age-Matched Control; CCC0, Cross-Covariance Coefficients; FOG, freezing of gait; IRP, Instantaneous Relative Phase.

Phase shift has been used as a metric to assess interlimb coordination in PD research. Two studies have examined phase shift to quantify the timing relationships between limb movements (Carpinella et al., 2007; Crenna et al., 2008). Carpinella et al. (2007) investigated phase shift changes and reported that reductions in phase shift were associated with improved interlimb coordination (Carpinella et al., 2007). This study also examined the effects of combining sensory and motor tasks but found no additional improvements in phase shift or range of motion. Similarly, Crenna et al. (2008) assessed phase shift between the arm and ipsilateral leg motion, noting significant reductions when PD patients were on medication, suggesting that pharmacological intervention may influence coordination patterns (Crenna et al., 2008). Moreover, Crenna et al. (2008) further analyzed phase shift by examining the rhythmic oscillations of limb movements during gait cycles in individuals with PD (Crenna et al., 2008). The study reported that a significant portion of PD patients exhibited irregular or absent arm swinging. Among those who demonstrated arm swing, the movement was primarily restricted to one cycle per stride, differing from the more variable oscillations observed in control participants. The study also assessed the effects of medication on phase shift, reporting changes in coordination patterns when patients were in the medicated state.

The Phase Coordination Index (PCI) has been used to assess interlimb coordination in individuals with PD. Three studies have examined PCI as a metric for quantifying bilateral coordination deficits (Martínez et al., 2018; Nanhoe-Mahabier et al., 2011; Peterson et al., 2012). Peterson et al. (2012) investigated PCI across different gait tasks and reported that individuals with PD, particularly those with freezing of gait (FOG), exhibited higher PCI values compared to PD patients without FOG and control participants (Peterson et al., 2012). Compared to age-matched controls, PD participants showed significantly elevated PCI scores, indicating impaired synchronization and phase control. Higher PCI values were observed during more complex gait tasks, such as turning. The study also examined the relationship between PCI and freezing severity, using FOG-Q scores to assess the extent of coordination impairments. Moreover, Martínez et al. (2018) examined PCI by analyzing its relationship with clinical and demographic variables, such as disease duration, motor symptom severity, and functional mobility (Martínez et al., 2018). The study assessed how PCI measurements varied across individuals with different levels of impairment, providing additional context for its use as a coordination metric. Their findings highlighted associations between PCI and multiple factors related to gait and mobility in PD.

### 3.7 Methodological quality assessment

The results of the risk of bias assessment are presented in Table 4. Overall, the methodological quality of the included studies was moderate to high with all studies providing clearly statements of the research objectives, outcome measures, and participant characteristics. Most studies provided estimates of random variability and reported appropriate use of statistical tests, supporting the reliability of their findings. However, important limitations were revealed from the Downs and Black checklist in that 10 of the 14 studies did not adequately describe the distribution of potential confounders, and 13 failed to report on adverse events (Downs and Black, 1998). Additionally, no study reported blinding of participants and outcome assessor blinding was unclear in 13 studies, leading to an increased risk of reporting and selection biases. Additionally, while probability values were consistently reported, adjustments for differences in follow-up durations were universally absent. Recruitment procedures were inconsistently reported with 7 out of the 14 studies marked as ‘unclear’ regarding whether samples were representative of the target population, and two studies were explicitly marked as ‘not representative.’ These findings highlight areas for improvement in methodological reporting (particularly regarding confounders, blinding, and adverse event disclosure) while also recognizing that many studies demonstrated strengths in outcome reporting and statistical analysis.

**Table 4:** Appraisal of studies using downs and black risk of bias assessment.

Assessment criteria	Carpinella et al. (2007)	Crenna et al. (2008)	Nanhoe-Mahabier et al. (2011)	Huang et al. (2012)	Peterson et al. (2012)	Rodriguez et al. (2013)	Roemmich et al. (2013)	Tanahashi et al. (2013)	Lin and Wagenaar (2018)	Martínez et al. (2018)	Filippin et al. (2020)	Agurto et al. (2021)	Arippa et al. (2022)	Mainka et al. (2023)
Clear hypothesis/ aim/objective	+	+	+	+	+	+	+	+	+	+	+	+	+	+
Clear outcome measures	+	+	+	+	+	+	+	+	+	+	+	+	+	+
Patient characteristics described	+	+	+	+	+	+	+	+	+	+	+	+	+	+
Interventions clearly described	+	+	+	+	+	+	+	+	+	+	+	+	+	+
Distributions of confounders described	−	?	?	+	+	−	+	+	+	+	+	?	+	−
Findings clearly described	+	+	+	+	+	+	+	+	+	+	+	+	+	+
Estimates given of random variability	+	+	+	+	+	+	+	+	+	+	+	+	+	+
Adverse events reported	−	−	+	−	−	−	−	−	−	−	−	−	−	−
Patients lost to follow-up described	×	×	×	×	×	×	×	×	×	×	×	×	×	×
Probability values reported	+	+	+	+	+	+	+	+	+	+	+	+	+	+
Recruitment pool represents population	−	?	?	?	?	?	+	+	+	+	+	+	?	?
Participants represent population	−	?	?	?	?	?	+	+	+	+	+	+	?	?
Staff/places/ facilities match standard treatment	−	−	−	−	−	−	−	+	−	+	−	+	?	+
Participants blinded to intervention	×	×	×	×	×	×	×	×	×	×	×	×	×	×
Those measuring outcomes blinded	?	?	?	?	?	?	?	?	?	?	?	−	?	?
Data dredging reported	−	−	−	+	−	+	+	−	−	+	−	?	−	?
Adjusted for different lengths of follow-up	×	×	×	×	×	×	×	×	×	×	×	×	×	×
Appropriate statistical tests	+	+	+	+	+	+	+	+	+	+	+	+	+	+
Reliable compliance with intervention	×	×	×	×	×	×	×	×	×	×	×	×	×	×
Main outcome measures used accurate	+	+	+	+	+	+	+	+	+	+	+	+	+	+
Patients in different intervention groups	+	+	+	+	+	+	+	+	+	+	+	+	+	+

Table format modified from Diment et al. (2018). “+” Yes; “₋” No; “?” Unsure; “×” Not applicable.

## 4 Discussion

This scoping review examined the impact of PD on interlimb coordination during gait and observed key differences between PD and older adults, highlighting the critical alterations associated with PD-related motor control impairments. Notably, gait dysfunction in PD manifests not only in variations of self-selected walking speed and spatiotemporal parameters, but also with altered interlimb coordination. One limitation identified from the findings is the lack of consistency in the methodological approaches across studies, particularly in terms of specific interlimb coordination metrics and varying protocols associated with to data collections. By synthesizing current evidence, this review clarifies the distinct interlimb coordination impairments associated with PD and highlights critical methodological gaps—laying the groundwork for future research to develop targeted, coordination-focused interventions that enhance mobility and reduce fall risk in this population.

### 4.1 Gait parameters

Spatiotemporal gait parameters in PD reflect fundamental impairments in locomotor control with key deficits in walking speed, stride length, cadence, and variability. These abnormalities stem from basal ganglia dysfunction, leading to impaired motor planning, execution, and adaptability (Huang et al., 2012; Nanhoe-Mahabier et al., 2011). Notably, reduced walking speed and stride length, commonly observed in PD, correlate with diminished ROM in the hip, knee, and upper limb joints, emphasizing the interconnected nature of spatiotemporal and ROM deficits in gait dysfunction. For instance, Carpinella et al. (2007) and Crenna et al. (2008) reported that PD patients exhibit significantly lower arm and thigh ROM, which worsens at higher walking speeds, suggesting that motor stiffness and rigidity restrict movement adaptability.

Building on the association between reduced walking speed and limited joint motion, subsequent research has shown that these restrictive movement patterns are further provoked under increased task demands. Specifically, studies have demonstrated that individuals with PD exhibit progressively reduced ROM as walking speed increases—suggesting a compounding effect of motor rigidity and impaired adaptability (Arippa et al., 2022; Carpinella et al., 2007; Filippin et al., 2020; Mainka et al., 2023; Roemmich et al., 2013). These limitations are primarily attributed to cardinal PD symptoms such as axial rigidity and bradykinesia. Axial rigidity restricts normal segmental rotations and leads to stiffness in the torso and hips, thereby reducing the natural range of motion during gait. Bradykinesia further exacerbates ROM restrictions by limiting the amplitude and fluidity of limb swings, especially at faster walking speeds (Lin and Wagenaar, 2018). While pharmacological treatments like L-DOPA and surgical interventions may partially improve these impairments, they often fail to restore ROM to normative levels (Buckley et al., 2017). In addition to these changes, some ROM reductions reflect compensatory strategies aimed at increasing postural stability and minimizing tremor or freezing episodes (Arippa et al., 2022; Carpinella et al., 2007; Crenna et al., 2008; Mainka et al., 2023; Roemmich et al., 2013). Although such adaptations may facilitate basic mobility, they often introduce increased gait asymmetries that, over time, undermine functional independence and elevate fall risk (Mainka et al., 2023; Plotnik et al., 2007). Overall, these findings underscore the importance of combining pharmacological and rehabilitative strategies to address both the primary motor impairments and the maladaptive compensations that limit ROM.

### 4.2 Coordination

The impact of PD on interlimb coordination profoundly influences gait adaptability, reflecting the complex interplay of neural and biomechanical impairments that disrupt the spatial and temporal organization of movement. Individuals with PD demonstrate significant alterations in interlimb coordination that includes phase shifts and synchronization delays as compared to young, healthy individuals (Carpinella et al., 2007; Crenna et al., 2008; Lin and Wagenaar, 2018; Martínez et al., 2018). These disruptions signify PD-related motor dysfunction, where impairments in neuromuscular control and motor planning lead to reduced gait stability and increased fall risks.

Several interconnected mechanisms likely underlie the coordination deficits observed in PD. The degeneration of the basal ganglia disrupts the central locomotor pattern generator, impairing the automatic, rhythmic control of gait and leading to irregular timing of strides (Huang et al., 2012; Martínez et al., 2018; Nanhoe-Mahabier et al., 2011). Additionally, typical PD symptoms restrict trunk rotation and slow movement execution, thereby further exacerbating phase shifts and synchronization delays during walking (Cole et al., 2017; Crenna et al., 2008; Dietz, 2011). Reduced sensitivity of leg extensor load receptors impairs the detection of ground reaction forces necessary for smooth gait cycles, likely also contributing to deficits (Martínez et al., 2018). Together, the disrupted phase relationships between arm and leg movements shifts individuals away from the desired anti-phase patterns observed in healthy individuals (Carpinella et al., 2007; Huang et al., 2012).

Environmental context also significantly impacts interlimb coordination in PD. For example, treadmill walking—by providing consistent rhythmic pacing and continuous visual flow—offers external cues that may help bypass impaired internal timing mechanisms associated with basal ganglia dysfunction, thereby supporting more consistent stride timing and enhanced interlimb synchronization (Lin and Wagenaar, 2018; Nanhoe-Mahabier et al., 2011). In contrast, overground walking demands greater adaptability and supraspinal control due to the absence of external pacing and the need for continuous self-regulation, which may reveal coordination impairments that are otherwise masked during treadmill walking. This was evident in studies where PD individuals demonstrated greater stride time asymmetry and increased PCI values during overground walking compared to treadmill conditions, particularly in those with freezing of gait (Nanhoe-Mahabier et al., 2011; Peterson et al., 2012). Thus, treadmill-based assessments may underestimate the severity of coordination deficits and lack the necessary ecological validity to capture the extent of motor dysfunction found with PD (Hafer and Boyer, 2018; Nanhoe-Mahabier et al., 2011).

Various coordination metrics—such as phase shift, synchronization delays, cross-correlation, and PCI—have been used to assess interlimb coordination deficits in Parkinson’s disease. Alterations in these metrics reflect impaired temporal coupling between limbs, which can manifest as difficulty executing complex gait tasks including turning, obstacle avoidance, and adapting to dynamic environments (Peterson et al., 2012). These coordination deficits are critically significant, as they contribute to increased fall risk, reduced mobility, and limited participation in daily activities (Kraan et al., 2017; Nanhoe-Mahabier et al., 2011). Some coordination metrics have also demonstrated sensitivity to dopaminergic therapy, with improvements observed following levodopa administration (Martínez et al., 2018), suggesting their potential utility in monitoring treatment effects (Figure 1).

**Figure 2:**
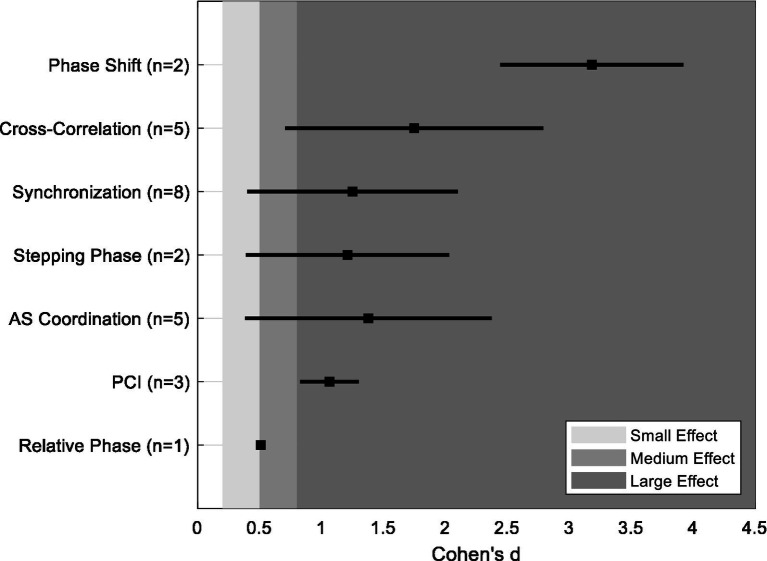
Computed mean and range of effects size (Cohen’s d) for each coordination metric.

To evaluate the ability of particular coordination metrics to distinguish between PD individuals and OA, we computed the overall mean and range of each coordination metric captured within the 14 studies of the scoping review. Figure 2 shows the effect size magnitude between PD and OA for individual coordination metrics. The majority of metrics exhibit large effects, although the range of values need to be noted as some metrics (synchronization, stepping phase, and cross-correlation) span across medium to large effect sizes. Conversely, the metric of relative phase showed small to medium effect sizes, suggesting limited sensitivity to differentiate PD and OA individuals. This comparative analysis needs to be viewed with caution due to the sample sizes used with the studies as well as with the limited number of data used to compute the mean effect sizes of the different coordination metrics. Future work that incorporates these variables can provide further insights when determining which coordination measures are most sensitive to disease-related changes and functional decline in PD.

### 4.3 Methodology

Assessing interlimb coordination and gait adaptability in individuals with PD relies on motion capture systems, accelerometers, and gyroscopes, with motion capture considered the gold standard (Carpinella et al., 2007; Crenna et al., 2008). However, the lack of standardized protocols across studies presents numerous challenges. The clinical heterogeneity associated with PD symptom severity, medication effects, and motor impairments necessitates stratification based on disease stage to enable meaningful comparisons (Agurto et al., 2021; Lin and Wagenaar, 2018).

Among the included studies, 13 out of 14 explicitly reported participants’ dopaminergic medication status. Most assessments were conducted in the “ON” medication state, typically 60 min to 12 h post-medication intake, while only one study (Tanahashi et al., 2013) assessed participants in the “OFF” state. In addition, clinical scores such as the Unified Parkinson’s Disease Rating Scale (UPDRS), Hoehn & Yahr (H&Y) staging, and the Mini-Mental State Examination (MMSE) were frequently used to contextualize participant status.

While the consistent reporting of medication status and clinical scores is a notable strength, the diversity of coordination metrics and limited sample sizes across studies remain major limitations, precluding subgroup analyses based on medication or disease severity. Future research should adopt standardized coordination metrics and larger, stratified samples to better elucidate how pharmacological status and disease progression influence coordination deficits in PD.

Environmental context also significantly influences gait outcomes. For instance, treadmill walking introduces rhythmic external cues that differ from the demands of overground walking (Caballero et al., 2019; Nanhoe-Mahabier et al., 2011). Moreover, the lack of consensus on key coordination metrics—such as phase shifts, synchronization delays, and PCI—complicates cross-study comparisons (Peterson et al., 2012; Roemmich et al., 2013). Varied marker sets, different approaches to data filtering, and lack of clarity around gait cycle definitions further contribute to reporting inconsistency (Carpinella et al., 2007; Mancini et al., 2021; Visscher et al., 2021). For example, the reviewed studies employed a range of motion capture systems (e.g., Vicon, Optotrak, inertial sensors), marker sets (ranging from 5 to 22 markers), and sampling frequencies (50–128 Hz). Filtering protocols also varied widely, with some studies applying low-pass Butterworth filters (cut-off frequencies between 3 and 10 Hz), while others did not report filtering parameters at all (Carpinella et al., 2007; Crenna et al., 2008; Filippin et al., 2020). Additionally, outcome measures were inconsistently defined, with studies reporting PCI, synchronization delays, and spatiotemporal parameters using non-unified analytical frameworks. These inconsistencies highlight the urgent need for standardized terminology, acquisition protocols, and processing pipelines to improve reproducibility and advance coordination research in PD.

### 4.4 Recommendations

To enhance the reliability and reproducibility of studies on PD and interlimb coordination, researchers should adopt standardized methodologies and detailed reporting. This approach would help address key sources of bias identified in the included studies (Table 4), such as insufficient documentation of confounding variables, lack of adverse event reporting, and absence of blinding procedures. Transparent reporting of these methodological aspects is critical to reducing inconsistencies and improving cross-study comparability.

Standardization of data acquisition procedures and signal processing protocols represent the strongest area of improvement for future studies on PD and coordination. Many studies used non-uniform pipelines for marker placement, filtering techniques, and gait event detection, which restricts reproducibility (Carpinella et al., 2007; Crenna et al., 2008; Filippin et al., 2020). Future work should prioritize the adoption of validated frameworks, which ensure consistency in motion capture and coordination quantification (Fukuchi et al., 2018). For example, adopting open-source toolkits such as the Gait and Balance Toolbox (Mancini et al., 2021) or validated protocols like those in the Brain Electrophysiological recording & Stimulation (BEST) toolbox (Hassan et al., 2022) can further strengthen reproducibility in coordination research.

Wearable sensors (e.g., IMUs, accelerometers) and machine learning algorithms represent emerging technologies that offer opportunities to evaluate interlimb coordination in both laboratory and naturalistic settings (Agurto et al., 2021). While traditional motion capture systems remain the gold standard due to their high spatial accuracy, they are limited to controlled environments. In contrast, wearable sensors enable gait monitoring in real-world contexts, but pose limitations due to the vulnerability to signal noise, placement variability, and calibration challenges. This trade-off between laboratory-based precision and ecological validity underscores the need to validate wearable technology against motion capture benchmarks and to develop standardized algorithms for extracting coordination metrics.

Additionally, future studies should address the ecological limitations of current research. Most assessments focus on straight-line walking in constrained laboratory environments (e.g., treadmill or overground), which do not capture the full complexity of everyday mobility. Researchers should incorporate more ecologically valid tasks—such as turning, dual tasking, and navigating environmental obstacles—to better characterize coordination deficits in PD. Additionally, longitudinal studies are also limited, yet essential insights can be obtained for tracking disease progression, identifying early indicators of gait deterioration, and developing preemptive interventions.

## 5 Conclusion

This scoping review underscores the significant impact of Parkinson’s disease (PD) on interlimb coordination, characterized by disrupted rhythmicity, synchronization, and motor adaptability—factors that contribute to gait instability. Despite consistent findings, methodological heterogeneity in measurement techniques, outcome metrics, and medication status reporting limits cross-study comparability. While treadmill-based assessments offer controlled conditions, the lack of ecological validity in real-world settings remains a critical gap. Advancing coordination research in PD requires the adoption of standardized metrics, longitudinal designs, and validated wearable sensors to enhance clinical relevance. These insights can guide personalized gait interventions and support the development of rehabilitation strategies aimed at improving mobility and monitoring disease progression in PD.
